# Sign Language Recognition Using the Electromyographic Signal: A Systematic Literature Review

**DOI:** 10.3390/s23198343

**Published:** 2023-10-09

**Authors:** Amina Ben Haj Amor, Oussama El Ghoul, Mohamed Jemni

**Affiliations:** 1 Research Laboratory LaTICE, University of Tunis, Tunis 1008, Tunisia; benhajamor.amina@gmail.com; 2 Mada—Assistive Technology Center Qatar, Doha P.O. Box 24230, Qatar; oelghoul@mada.org.qa; 3 Arab League Educational, Cultural, and Scientific Organization, Tunis 1003, Tunisia

**Keywords:** sign language recognition, systematic review, sEMG, electromyographic signal

## Abstract

The analysis and recognition of sign languages are currently active fields of research focused on sign recognition. Various approaches differ in terms of analysis methods and the devices used for sign acquisition. Traditional methods rely on video analysis or spatial positioning data calculated using motion capture tools. In contrast to these conventional recognition and classification approaches, electromyogram (EMG) signals, which measure muscle electrical activity, offer potential technology for detecting gestures. These EMG-based approaches have recently gained attention due to their advantages. This prompted us to conduct a comprehensive study on the methods, approaches, and projects utilizing EMG sensors for sign language handshape recognition. In this paper, we provided an overview of the sign language recognition field through a literature review, with the objective of offering an in-depth review of the most significant techniques. These techniques were categorized in this article based on their respective methodologies. The survey discussed the progress and challenges in sign language recognition systems based on surface electromyography (sEMG) signals. These systems have shown promise but face issues like sEMG data variability and sensor placement. Multiple sensors enhance reliability and accuracy. Machine learning, including deep learning, is used to address these challenges. Common classifiers in sEMG-based sign language recognition include SVM, ANN, CNN, KNN, HMM, and LSTM. While SVM and ANN are widely used, random forest and KNN have shown better performance in some cases. A multilayer perceptron neural network achieved perfect accuracy in one study. CNN, often paired with LSTM, ranks as the third most popular classifier and can achieve exceptional accuracy, reaching up to 99.6% when utilizing both EMG and IMU data. LSTM is highly regarded for handling sequential dependencies in EMG signals, making it a critical component of sign language recognition systems. In summary, the survey highlights the prevalence of SVM and ANN classifiers but also suggests the effectiveness of alternative classifiers like random forests and KNNs. LSTM emerges as the most suitable algorithm for capturing sequential dependencies and improving gesture recognition in EMG-based sign language recognition systems.

## 1. Introduction

The use of body movements as a nonverbal means of interaction is a powerful communication tool. While hearing individuals use gestures to complement their speech, deaf people rely on them as the foundation of their language. Therefore, sign language constitutes their primary mode of communication. It is important to note that deafness is a unique disability that remains hidden, and due to hearing loss, it excludes deaf and hard-of-hearing individuals from accessing auditory information available to the hearing population. Consequently, deaf individuals face a significant information deficit.

To address this disparity, various initiatives have been implemented to make public information accessible to deaf individuals. The prospect of creating real-time translation systems between spoken languages and sign languages is intriguing, especially considering that sign language is used by a minority of hearing individuals. Recognizing the gestures used by deaf individuals in their communication process is a crucial step towards achieving this goal and promoting accessibility and social integration within this community. However, research in the field of automatic sign language recognition is limited. Most research relies on motion capture and gloves, images, videos, and, more recently, sEMG data. Despite this, sign language recognition methods are continually evolving, with ongoing refinements in computer vision methods and exploration of various sensor-based approaches. 

With technological advancements, several complex devices and sensors have been developed for use in sign language recognition. Initially, participants wore colored gloves to simplify the recognition task. However, more recent systems, based on vision and electromyogram (EMG) sensors, offer potential technology for gesture recognition. These systems have resulted from extensive research and effective marketing efforts, including the active and passive gloves, leap motion, and sEMG sensors. sEMG-based systems were initially employed in various applications and experiments during the 1980s, with several researchers utilizing them for data acquisition. The acquisition of the EMG signal is a crucial step in ensuring the effectiveness of the sign language recognition system [1], distinguishing it from classical sign language recognition approaches [2]. sEMG data can be obtained from several existing wearable devices in the market, including a set of EMG sensors that can be designed as wristbands worn by signers. Several significant parameters influence the analysis of EMG signals, including the quality of the contact between the electrode and the skin, skin properties, muscle energy state, and the distance between the sEMG sensors [3,4]. Additionally, the type and size of the sEMG sensors directly affect the bandwidth and amplitude of the signal. It is worth noting that EMG signals typically possess a bandwidth ranging from 10 Hz to 10,000 Hz and an amplitude ranging from 10 μV to 5 mV.

This systematic review aimed to assess the current state of research on using EMG signals for sign language recognition. This review systematically searched and evaluated the relevant literature, examined data acquisition methods and their impact, analyzed feature extraction and classification techniques, assessed dataset relevance, and evaluated participant diversity in studies. It analyzed feature extraction and classification techniques applied to EMG signals and identified the most used datasets, evaluating their relevance and suitability. This review also assessed the current state of research considering sample size and participant diversity in studies, providing a summary and making recommendations for future research. Additionally, it addressed the challenges and limitations, including issues related to feature extraction and classification, of using EMG signals for sign language recognition. This review summarized and discussed 88 selected studies on sign language recognition using EMG signals. Chinese sign language is the most frequently studied, with 25% of the reviewed studies focusing on it. American sign language is the second most studied. This review sheds light on the use of EMG signals in sign language recognition research and the prevalence of studies on specific signed languages. 

The paper is organized as follows: In Section 2, we provide a comprehensive exploration of various essential aspects of our research. This section begins by presenting the background and objectives that underpin our study. We delved into the context and motivations that drove our investigation. Moving forward, the subsequent section is dedicated to describing the techniques and devices employed in the acquisition of data. Here, we meticulously examined the devices and methodologies used to capture the EMG signals. This section serves as a basis for understanding the data gathering process and its implications on the subsequent stages of our analysis. Following our exploration of data acquisition, we pivot to a section focused on the techniques used in the extraction of features and the processing of data. In this part of our study, we presented the methods used to derive meaningful information from the collected EMG signals. In Section 5, we transition to another section that spotlights the classification approaches most frequently employed in the domain of EMG-based sign language recognition. Here, we delved into the algorithms and methodologies used to categorize and identify sign language gestures from the processed data. This section offers valuable insights into the classification techniques that have been pivotal in advancing the field. In the Section 6, we enter a detailed discussion aimed at comparing different approaches and devices used for data acquisition. Within this section, our primary goal was to extract meaningful findings by dissecting the algorithms, methodologies, and tools employed in categorizing and identifying sign language gestures from the processed data. Additionally, we engaged in a comparative analysis to evaluate accuracy and performance.

## 2. Background and Objectives

In this section, we provide an overview of the background and the fundamental objectives that underpin the entire review related to EMG signal acquisition and processing for sign language recognition. We thoroughly explore the contextual landscape of EMG signal acquisition and processing, elucidating the key factors and motivations driving our survey.

### 2.1. Background

Video, motion capture (mocap), and surface electromyography (sEMG) are distinctive technologies employed for sign language recognition, each offering unique advantages and facing different challenges (Table 1). Video-based recognition is versatile, dealing with 2D/3D visual data, offering high resolutions and moderate portability at a moderate cost. It is, however, sensitive to light conditions. Mocap provides high-resolution 3D motion analysis but is less portable and more expensive, with sensitivity to marker occlusions. sEMG, capturing muscle activation, stands out for its portability and ability to record static and dynamic gestures, making it valuable for muscle analysis in sign language. Each technology, with its unique attributes, serves varied applications in sign language recognition.

Electromyogram (EMG) signals are biological signals corresponding to the electric current captured on the skin surface near the muscle during its contraction [5]. They are controlled by the nervous system. In fact, electromyography (EMG) [6] processes neuromuscular activity and muscle morphology by measuring muscle responses or electrical activity produced by skeletal muscles. EMG signals concern two types of electrical activity of the motor units of a muscle:

- The first type is the surface EMG (sEMG), which is recorded via non-invasive electrodes, often used to obtain data on the intensity of the superficial muscle activation or on the time [7].
- The second is the intramuscular EMG that is recorded via invasive electrodes [8].

**Figure 1:**
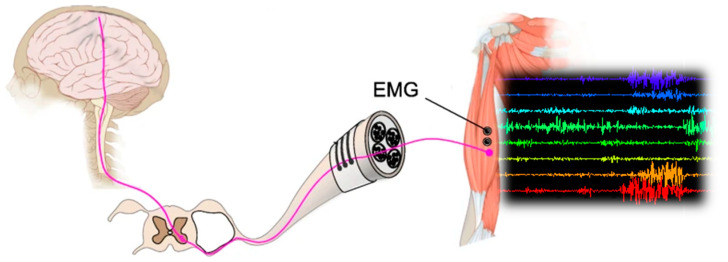
The EMG signal generation principle.

The electrical current generated in a muscle during the contraction or detraction represents neuromuscular activities, measured via EMG signals [9], as shown in Figure 1. This can be used for various applications. Electromyography (EMG) studies neuromuscular activity and muscle morphology by measuring muscle responses or electrical activity produced by skeletal muscles. The nerves control muscles through electrical signals called impulses. These impulses are measured and recorded using EMG sensors via surface electrodes, where the electrodes are placed directly on the muscle to detect muscle impulses. Indeed, to generate muscle movements, electrical signals are transmitted from the brain to one or more neurons. Each neuron is attached to hundreds or even thousands of muscle fibers, causing them to contract while the neuron(s) remain active [10]. The summation of the electrical activity is produced by the neurons and contraction of all affected muscle fibers. The latter is called the motor unit potential (MUP), which contributes to the signal generation and gives a stochastic nature to EMG [11].

**Figure 2:**
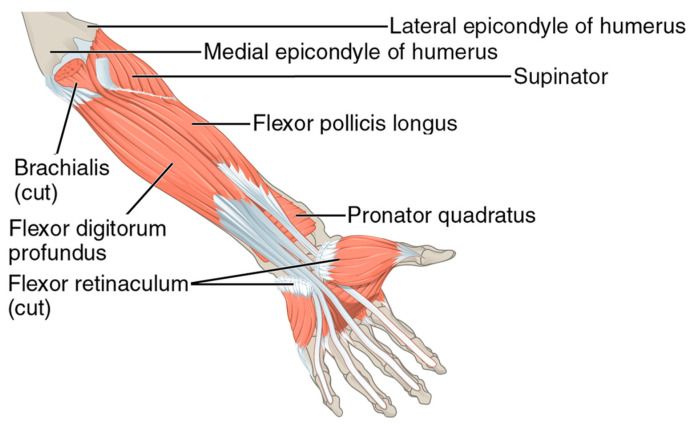
Arm muscles.

**Figure 7:**
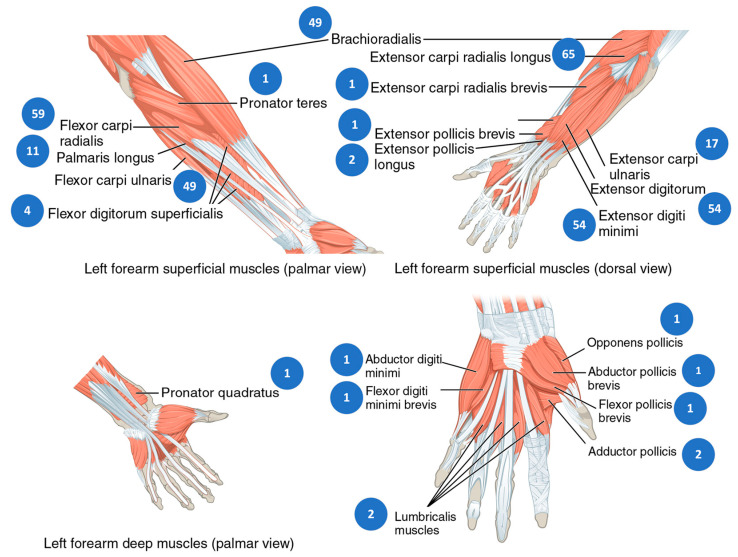
Number of Papers Targeting Each Muscle During Data Collection.

Our work has discussed papers that primarily focus on the movement of the hand, specifically controlled by the extrinsic muscles, which originate in the forearm and extend into the hand, and the intrinsic muscles, which are located entirely within the hand. Figure 2 and Figure 7 depict these muscles.

Electromyogram (EMG) signals offer significant potential for technical applications, as they provide detailed information on muscle activity across different scales. EMG signals are widely used to study human body behavior, and their recording from muscles presents an opportunity to identify various characteristics. However, the complex patterns of EMG signals and the noise they generate, particularly during movement, make their classification challenging. Nevertheless, advancements in signal processing and machine learning techniques are making the use of sEMG signals in domains, such as the human–computer interaction, a promising research area.

Research on the domain of the human–computer interaction (HMI) represents a relatively new area of interest. In this domain, electromyographic (EMG) signals have been utilized for a variety of clinical and industrial applications, such as controlling exoskeletons, robotic prostheses, wheelchair robotics, and hand gesture recognition applications. These applications rely on the classification of EMG signals via electromyographic control techniques. However, the accurate acquisition of the EMG signal is a crucial step to ensure the efficacy of these applications. Over the past few decades, numerous researchers have shown a keen interest in utilizing surface electromyography (sEMG) signals for hand gesture recognition, and for this purpose, several features can be extracted from these signals. However, the sEMG signals are characterized by high variability and complexity, thus necessitating the extraction of a vast amount of information for feature extraction. In order to recognize and analyze hand gestures, a series of quantitative measurements must be analyzed, broken down, and classified.

This review was concerned with the classification of electromyographic (EMG) signals for the purpose of sign language recognition [12]. The recognition of sign language is of great significance, as it allows people with hearing or speech impairments to effectively communicate with others. EMG signals have shown promising results for the recognition of sign language due to their ability to capture the electrical activity of muscles that control hand movements and gestures.

### 2.2. Objectives

This systematic review was conducted in accordance with the Preferred Reporting Items for Systematic Review and Meta-Analysis protocol [13]. To begin, we formulated the research questions, which have been described on the subsequent page, followed by the development of a search strategy. 

**Figure 3:**
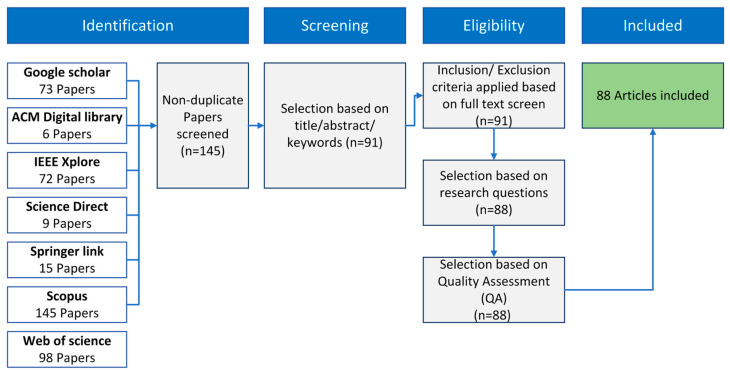
Review steps.

Figure 3 illustrates the process we employed to select relevant papers. Initially, we conducted searches through seven web platforms, as specified in the identification step of Figure 3. We sought papers containing the term “sign language,”, along with “recognition” or “classification,”, and at least one of the following terms: “emg,”, “semg,”, “electromyography,”, or “electromyographic” within the title, abstract, or keywords. Employing these criteria yielded a set of 418 papers. After removing duplicates, we secured a total of 123 unique articles for further screening. Each article underwent a rigorous evaluation to assess its relevance and quality within the context of our research. The screening step involved reviewing abstracts, titles, and keywords to select papers that utilized sEMG signals for sign language recognition, leaving us with 91 significant papers out of the 123 initially selected during the screening step. After the screening step, selection through eligibility criteria was applied. We selected papers that focus on the recognition of static or dynamic signs in sign languages. Papers that used mixed approaches, like the combination of sEMG and inertial data, were also considered eligible. The quality of the included studies was subsequently assessed using specific criteria. We only retained papers that provided information about the utilized dataset, such as its size, the number of subjects, and the data collection methodology. Additionally, we only included papers that clearly presented the employed approach and reported the accuracy obtained with that approach. Finally, 88 papers were included in the review.

The results of the systematic review were then synthesized and interpreted, considering the strengths and limitations of the included studies. The systematic review process ensures a rigorous and transparent approach to synthesizing the available evidence, providing a comprehensive and reliable overview of the existing literature.

The goal of this systematic review was to take a closer look at the current state of research on using EMG signals to recognize sign language. This review carefully examined the relevant studies and research in this field. We also looked at the methods and tools used to capture EMG signals and how they impacted the recognition results.

This review has:

1. Systematically searched, identified, and critically evaluated the relevant literature on sign language recognition using EMG signals.
2. Investigated the various data acquisition methods and devices used to capture EMG signals and their impact on the recognition performance.
3. Analyzed the different feature extraction and classification techniques applied to EMG signals for sign language recognition.
4. Identified the most used datasets and evaluated their relevance and suitability for sign language recognition using EMG signals.
5. Assessed the current state of research in terms of the sample size and the diversity of the participants in the studies.
6. Provided a summary of the current state of research on sign language recognition using EMG signals and made recommendations for future research.
7. Identified the challenges and limitations of using EMG signals for sign language recognition, including problems related to signal quality, feature extraction, and classification.

This review has presented a comprehensive summary and critical discussion of 88 selected studies that focused on sign language recognition using electromyographic (EMG) signals. The reviewed articles covered research on 21 distinct sign languages, and our systematic analysis revealed that Chinese sign language was the most frequently used language for EMG-based classification works. Specifically, 21 out of the 88 studies (24%) in our review focused on Chinese sign language, with an equal number of papers dedicated to American Sign Language. Our review highlights the utilization of EMG signals in sign language recognition research and provides insights into the prevalence of research on specific signed languages. Table 2 provides an overview of the commonly used sign languages and their corresponding percentages of use, as well as references to the studies that employed EMG signals for sign language recognition.

## 3. Data Acquisition and Devices

In this section, we will discuss various aspects of the data acquisition process for sign language recognition systems. Specifically, we will focus on the devices and sensors used to collect gesture and motion data, the number of subjects involved in data collection, and the environmental conditions under which the data were acquired. These factors play a crucial role in determining the quality and quantity of data available for training and testing recognition algorithms. The selection of devices and sensors used in data acquisition is an important consideration when developing sign language recognition systems. The choice of sensors may depend on certain factors, such as the desired level of detail, the cost, and complexity of the sensor setup. Moreover, the number of subjects involved in data collection is also an important factor to consider. Larger datasets can improve the generalizability of recognition algorithms and provide a more representative sample of the population. However, collecting data from a large number of subjects can be time-consuming and may require additional resources. Lastly, environmental conditions, such background noise, and movement restrictions can affect the quality of data collected. Therefore, efforts have been made to control these factors during data acquisition to ensure that the collected data is of high quality and suitable for use in training and testing recognition algorithms.

Before we look at how these data are collected and what devices are used in the selected studies, we will begin by showing the types of gestures that have been studied. We will give a detailed overview of the specific gestures mentioned in these studies.

**Figure 4:**
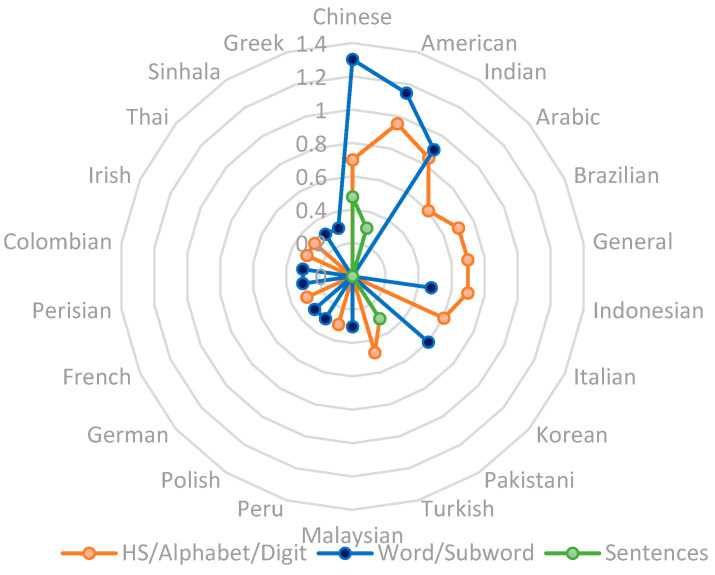
Categories of recognized gestures by sign language.

Figure 4 displays the types of gestures recognized in sign language. We have identified three distinct categories of these gestures. The first category, static gestures, covers hand shapes, alphabet letters, and digits. The second, dynamic gestures, includes words and subwords. Lastly, there is a category dedicated to sentences. Notably, the sentence category is the least represented among the three. We found that this category was only used on three languages: Chinese, American, and Pakistani. Furthermore, for more than half of the sign languages, we only noted one paper addressing sign language recognition using EMG data. For the American, Chinese, and Indian sign languages (the three most frequently encountered languages), we observed that dynamic gestures are more commonly identified. However, for the other languages, static gestures tended to be more common.

**Figure 5:**
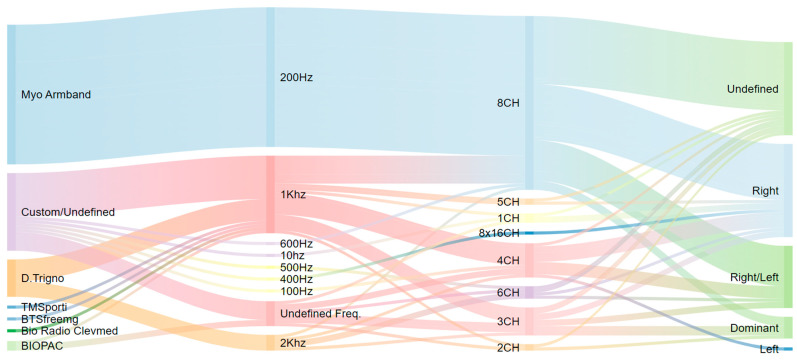
Data acquisition devices.

Figure 5 presents an overview of the studies undertaken between 2010 and 2022 that employed surface electromyography (sEMG) data to recognize sign language gestures; more detailed information is available in Table A1 of the Appendix A. These studies have utilized sEMG sensors to capture the electric signal data corresponding to gestures. Positioned on the forearm’s skin surface, sEMG sensors record the electrical activity manifested by the muscles in motion, serving as a direct indicator of muscular activity. Generally affixed to the user’s hand, wrist, or forearm, these sensors acquire data amidst the execution of sign language gestures. The integration of sEMG with IMU data or alternative data types, such as leap motion, has been proven to elevate recognition accuracy and fortify the identification of complex gestures. A review of the findings collated in Table A1 offers valuable insights into the efficacy of various data gathering and analysis methods in the field of sign language recognition.

This review revealed that the Myo armband is the most commonly used device in EMG-based sign language recognition systems (Figure 5). This is due to its affordability compared to other devices. Custom devices were found to be the second most commonly used after Myo, representing a significant proportion of the devices used in the studies we reviewed. Together, Myo and custom devices accounted for about 80% of the devices used in the reviewed studies. Non-Myo armband devices were found to operate at a frequency of 1 kHz, which is higher than the 200 Hz used in Myo armband devices. Additionally, we found that eight-channel devices were the most commonly used. These findings suggest that the Myo armband is popular due to its cost-effectiveness, while custom devices offer more flexibility and customization options. The dominance of the Myo armband and custom devices in the reviewed studies highlights the importance of considering device specifications when developing EMG-based sign language recognition systems. Specifically, the frequency and number of channels must be carefully selected to ensure optimal system performance [3].

In addition to the previously mentioned findings, we also noted that the majority of the reviewed studies only used one hand, either the right or dominant hand, for EMG-based sign language recognition. Out of the 88 reviewed studies, only 20 studies used data from both hands. This finding highlights the need for further research in using both hands simultaneously in EMG-based sign language recognition systems. It is possible that using data from both hands could increase the accuracy and efficiency of such systems, especially in recognizing complex signs and sentences that require coordinated movements of both hands. Therefore, future studies may consider exploring the use of both hands in EMG-based sign language recognition systems.

The accuracy of recognition varies in different studies due to certain factors, such as the complexity of gestures, type of data used, performance metrics, and devices used for data acquisition. Notably, approximately 50% of the studies employed the Myo armband from Thalmic Labs, which includes eight sEMG sensors for data acquisition. Figure 5 provides a visual representation of the frequency with which different EMG devices were utilized in the studies discussed in this article. This information can be useful in understanding the popularity and effectiveness of different devices for acquiring sEMG data.

Findings from the results obtained in the different studies examined in this article indicate that the use of the Myo armband can yield accurate results.. In the study published by the authors of [41], the researchers utilized two Myo armbands and assessed the performance of their approach by experimenting with different combinations of characteristics. Remarkably, they achieved 100% accuracy using surface electromyography (sEMG) in conjunction with accelerometer, gyroscope, and magnetometer sensors. Moreover, an accuracy rate of 99.25% was obtained using sEMG with accelerometer and magnetometer sensors. They also achieved an accuracy of 95.55% using the combination of sEMG, accelerometer, and gyroscope data, while the minimum accuracy of 47.4% was obtained when only sEMG data were used. This study emphasized the importance of combining different sensors to achieve high accuracy in measuring movement and orientation. The authors of [85] also obtained a high accuracy rate, equivalent to 99.6%, for the recognition of 30 gestures in Korean sign language, also using EMG and IMU data obtained from the Myo armband. According to our review of the literature, the best results were obtained via the approach that used the maximum number of sEMG sensors. In fact, the more the number of sEMG sensors is increased, the more the precision of the result is improved. In this context, so far, the device that contains the maximum number of sEMG sensors is the Myo armband.

**Figure 6:**
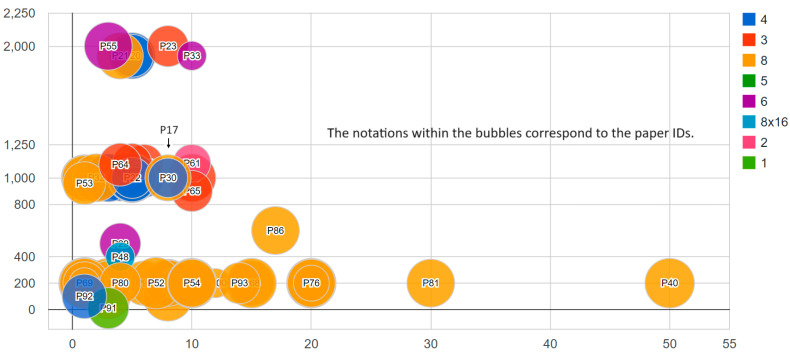
Subject number vs device frequency. X = subject number, Y = device frequency, bubble size = accuracy, and bubble color = number of channels.

From Figure 6, it is clear that the studies can be categorized into three distinct groups based on the frequency of their data frames. Specifically, these groups revolve around frequencies of approximately 200 Hz, 1000 Hz, and around 2000 Hz. The frequency appears to have no discernible impact on the accuracy. Many studies within the first frequency group achieved a higher accuracy compared to those in the second and third groups. In contrast to the frequency, the number of channels appears to significantly influence the accuracy. Both datasets [17,30] share a similar number of categories and utilize EMG, gyroscopes, and accelerometers for data collection. However, despite its significantly larger size, the dataset published by the authors of [30] yields lower accuracy compared to the dataset published by the authors of [17]. This discrepancy was attributed to the different number of channels used in each setup.

From examining Figure 6, it is evident that most studies utilized eight-channel EMG data, which represents the maximum number of channels used, excluding the study published by the authors of [48]. This study stands out for utilizing a high-definition device with an 8 × 16 channel matrix. In the study published by the authors of [48], the researchers achieved a 78% accuracy rate using a relatively compact database consisting of only 120 entries, spanning 4 subjects and 10 classes. When comparing the study published by the authors of [48] with other studies based on dataset size, two papers stood out due to their smaller dataset sizes and their exclusive reliance on EMG sensors. Both of the studies published by the authors of [63,92] achieved accuracies exceeding 90%, which is higher than that obtained by the authors of [48]. However, it is essential to approach these results with caution. The high accuracy rates achieved by the authors of [63,92] might be attributed to their tests being conducted on a single subject, while the study published by the authors of [48] tested on four subjects. The difference in subject count can significantly influence results, making direct comparisons less conclusive. A dedicated study focusing on the optimal number of channels is essential for determining the ideal configuration for obtaining the most accurate results.

The results obtained were related to several parameters, among which include the following parameters: the dataset and the number of subjects participating in the experiment. According to Table A2, the best result is obtained via the search conducted by the authors of [85], which achieved an accuracy of 99.6%, and the dataset used in this approach was collected from one subject. In the study published by the authors of [38], their experiments were conducted on a single subject; the observed accuracy rate was 80%, while the accuracy rate decreased to less than 34% for the multi-subject dataset. Figure 6 gives an overview of the number of subjects who participated in the experimentation of different approaches treated in this study; in most cases, the number of subjects was less than five. This study should also investigate the best placement locations for the sensors, as both the number and location can significantly impact the quality of the data and subsequent analysis.

Recognizing sign language through EMG signals poses a significant challenge due to the complex and diverse muscle movements involved in signing. Nevertheless, the identification of key muscles that play a crucial role in accurate recognition, such as those responsible for finger movements and wrist extension, is essential. Targeting these muscles using advanced machine learning algorithms to analyze their EMG signals has led to the development of highly accurate sign language recognition systems. There are several muscles in the forearm and hand that are responsible for finger movement and control. These muscles are divided into two groups: the extrinsic muscles, which originate in the forearm and extend into the hand, and the intrinsic muscles, which are located entirely within the hand. The extrinsic muscles involved in controlling finger movements can be divided into two groups: the flexor muscles and the extensor muscles. The flexor muscles responsible for finger flexion include the flexor digitorum profundus muscle, flexor digitorum superficialis muscle, and flexor pollicis longus muscle. On the other hand, the extensor muscles, namely the extensor digitorum muscle, extensor pollicis longus muscle, and extensor pollicis brevis muscle, play a key role in finger extension.

Finger control relies on a set of intrinsic muscles, namely the lumbrical muscles, interossei muscles, thenar muscles, and hypothenar muscles. The interossei muscles play a pivotal role in the abduction and adduction of the fingers, allowing for movements away from and towards the midline of the hand. On the other hand, the lumbrical muscles contribute to flexing the metacarpophalangeal joints and extending the interphalangeal joints of the fingers. These intricate actions are essential for performing precise finger movements, grasping objects with varying degrees of force, and executing intricate hand gestures with ease and finesse. The combined efforts of these intrinsic muscles facilitate our ability to manipulate our fingers with accuracy and control. The collective efforts of both the extrinsic and intrinsic muscles synergistically contribute to the fine motor control required for precise finger movements.

The exploration of finger muscles and their potential for sign language (SL) recognition has revealed intriguing patterns. Figure 7 illustrates how many papers targeted each muscle on the data collection phase. Notably, only one paper thoroughly investigated the intrinsic muscles that control finger movements, indicating a significant gap in our understanding and utilization of these muscles for SL recognition. Meanwhile, the extensor digitorum and flexor digitorum superficialis muscles have emerged as widely used and studied in this context, demonstrating promising results. However, it is important to acknowledge the untapped potential of other finger muscles. Leveraging the capabilities of these overlooked muscles presents a thrilling opportunity for further advancements in SL recognition using electromyography (EMG) signals. By harnessing the untapped potential of these muscles, we can potentially uncover new avenues for enhancing SL recognition and developing solutions to improve communication with the deaf community through sign language.

Data acquisition is a crucial step in sign language recognition using electromyography (EMG) signals. In this process, EMG sensors are attached to the muscles of the signer’s forearm to capture the electrical activity generated during sign language production. The EMG signals are then amplified, filtered, and sampled at a high frequency to obtain a reliable representation of the muscle activity. The resulting data are typically preprocessed by removing artifacts and noise, followed by feature extraction and selection to capture the relevant information about the sign language. Accurate data acquisition is critical to ensure the reliability and effectiveness of sign language recognition systems that use EMG signals. Several factors need to be considered to ensure accurate and reliable data acquisition. These include the selection of appropriate EMG sensors and placement of sensors on the appropriate muscle groups. The sampling frequency and filtering of the EMG signals also play a vital role in ensuring accurate data acquisition. Proper preprocessing techniques, such as noise removal and artifact removal, are critical to reducing interference and improving the quality of the EMG signals.

Studying the used datasets is another important aspect of the research. The dataset should contain a diverse range of sign language gestures, with sufficient examples for each gesture to ensure robust recognition accuracy. The number of classes in the dataset should correspond to the number of unique signs in the sign language. The choice of subjects is also critical to ensure that the dataset is representative of the population that will use the system. Subjects with different levels of sign language proficiency, hand sizes, and muscle strength should be included to ensure that the system is robust and can be used by a diverse range of users. The size of the dataset should be large enough to provide sufficient training examples for machine learning algorithms to achieve high recognition accuracy. The use of publicly available datasets can provide a benchmark for comparing different approaches and facilitating the development of sign language recognition systems. Table A2 provides a summary of the main factors involved in the process of building the dataset and acquiring data.

**Figure 8:**
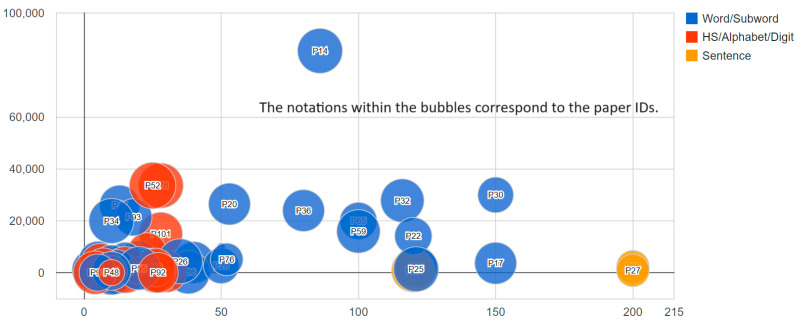
Class number vs dataset size. X = number of classes, Y = size of dataset, bubble size = accuracy, and bubble color = data type.

From Figure 8, we can observe that most of the studies use datasets with less than 40,000 entries. Additionally, the majority of these papers aimed to identify less than 50 distinct classes. The study published by the authors of [14] stands out as the sole study with a dataset size surpassing 40,000 entries, boasting 85,000 entries to discern 86 classes, and achieving an accuracy rate of approximately 94%. In contrast, the study published by the authors of [25] managed to attain an accuracy rate of 91% in identifying 121 classes with a significantly smaller dataset of roughly 1500 entries. Even though the study published by the authors of [14] incorporated EMG, along with accelerometers, gyroscopes, and magnetometers, the study published by the authors of [25] utilized just the EMG combined with accelerometers. We can conclude that while database size plays a significant role, achieving a high level of accuracy is still possible with reasonably sized databases.

Of the 88 papers reviewed, all papers chose to record their data afresh. This overwhelming preference for original data collection can be attributed to the inherent regional nature of sign languages. According to the *Ethnologue* report of 2023, there are over 159 official sign languages globally. Given this immense diversity, relying on a single predefined dataset may not provide a comprehensive or accurate representation of a specific sign language, as nuances and variations exist across regions. Furthermore, this trend can also be attributed to the fact that this research area is relatively recent. Newer fields often require primary data collection, as standardized datasets might not yet exist or the available datasets might not be extensive enough to encompass the depth and breadth of the topic under study. Thus, researchers often prioritize gathering fresh data that is more tailored to their study’s focus and region.

Upon reviewing the literature, we discovered six open datasets detailed in Table 3. Three of these datasets were centered on alphabet letters from the American, Italian, and Arabic sign languages. Meanwhile, another dataset emphasized individual words, and one was tailored towards sentences. Given the limited number of sign languages covered, the size of these datasets, the number of subjects involved, and the scope of vocabulary included, many researchers opted to create their own personalized datasets to better suit their specific research needs.

## 4. Feature Extraction

Feature extraction plays a vital role in developing a reliable sign language recognition system using electromyography (EMG) signals. The study published by the authors of [101,102] affirms the importance of meticulously describing the signals to execute an accurate classification. The feature extraction process involves identifying and extracting pertinent information from the raw EMG signals, which represent the electrical activity of muscles [103]. The extracted features include amplitude, frequency, and power spectrum, among others, to capture key characteristics. Similarly, in sign language recognition, feature extraction focuses on extracting relevant features from the hand and arm motions and shapes of the signer. These features are then utilized to classify signs and accurately interpret the intended message. Commonly employed techniques for feature extraction encompass time-domain analysis, frequency-domain analysis, and time-frequency analysis, all of which have proven effective in extracting meaningful features from signals. Proper feature extraction is crucial for accurate classification of hand gestures and ensuring precise recognition of signs. These features encapsulate various aspects of the EMG signal, such as magnitude and frequency, providing a concise representation that can be employed for classification. Once the appropriate features are extracted, a machine learning algorithm can be trained on these features to learn the relationships between the extracted features and the corresponding signs, resulting in enhanced accuracy and robustness.

The classification of EMG signals in sign language recognition has been a subject of extensive research, leading to the exploration of various types of features [104,105,106,107]. Table 4 presents a collection of commonly employed features for EMG-based sign language recognition. These features have been carefully selected and widely utilized in the literature to capture the relevant information from EMG signals and enable accurate classification. The diversity of these features reflects the different aspects of EMG signals that are informative for sign language recognition. By leveraging these features, researchers have been able to develop robust classification models capable of interpreting the intended signs with high accuracy. It is worth noting that the selection and combination of features may vary depending on the specific requirements of the sign language recognition system and the characteristics of the EMG signals under consideration.

Based on the insights obtained from Table 4, it can be observed that time-domain features are more frequently employed compared to frequency-domain features. Time-domain features are prominently favored due to their ability to capture important temporal characteristics and dynamics of the signals. These features offer valuable insights into the amplitude variations, temporal patterns, and timing information within the EMG signals. By emphasizing the time-domain features, researchers have been able to effectively extract relevant information related to muscle activation timing, muscle contraction duration, and temporal patterns associated with muscle activity. While frequency-domain features are also important for capturing spectral content and frequency-related characteristics, the prevalence of time-domain features in the literature suggests their significance in sign language recognition applications utilizing EMG signals. Although some comparative studies have indicated that frequency-domain features offer better performance in gesture classification, a specific study published by the authors of [108] explored thirteen different features from both the time domain and frequency domain of EMG signals for hand movements. The analysis revealed that features in the frequency domain exhibited superior dominance and signal characterization compared to features in the time domain. This suggests the potential advantages of utilizing frequency-domain features for accurate and effective gesture classification based on EMG signals. In addition, another research paper [109] further reinforced the superiority of frequency-domain features over time-domain features in terms of classification accuracy. The experimental results clearly demonstrated a significant improvement in the classification performance when utilizing frequency-domain features compared to time-domain features. This finding strengthens the notion that frequency-domain analysis provides valuable insights into the signal characteristics, enabling a more precise and reliable classification of signals.

**Figure 9:**
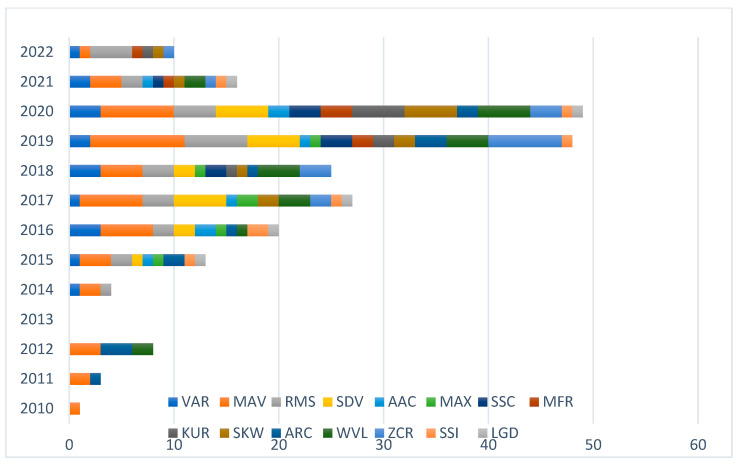
Evolution of the used features in EMG-based sign language recognition.

As illustrated in Figure 9, there has been a marked increase in the use of diverse features for sign language classification from EMG data, spanning the period from 2015 to 2022. Predominantly, time-domain features, such as variance, mean absolute value, and root mean square, were the most widely used between 2015 and 2021, underscoring their central role during this time frame. In addition, from 2020 to 2022, the employment of other features, including mean frequency, kurtosis, and skewness, became apparent, indicating a broadening in the array of features leveraged in this type of data analysis.

## 5. Classification Approaches

In this section, we present an overview of the methodologies and algorithms employed in the various studies examined during our comprehensive literature review. The classification of signs in sign language using EMG signals encompasses a range of techniques, predominantly machine learning and deep learning. These approaches leverage diverse classifiers when working with extensive signal datasets. By utilizing EMG, they achieved notable accuracy rates in classification. Selecting the most suitable algorithm relies on the specific task and available dataset, often requiring experimentation with multiple algorithms to identify the optimal choice for a given objective. Table A1 (Appendix A) provides a summary of all approaches discussed in relation to sign language recognition and sEMG signals. By providing more recent results, as well as important historical context, this table and the corresponding discussion provide insight into current trends in sign language recognition using sEMG signals.

**Figure 10:**
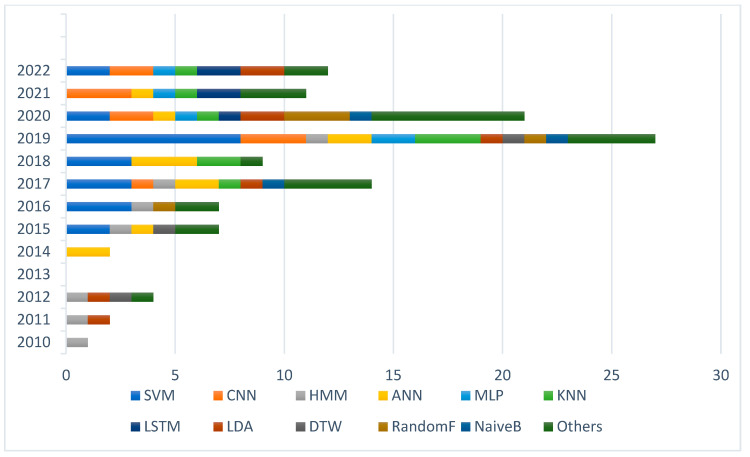
Evolution of approaches in EMG-based sign language recognition.

Based on the insights gathered from Figure 10, it is evident that various approaches have been employed for sign language recognition using electromyography (EMG) signals. Notably, between 2010 and 2014, hidden Markov models (HMMs) and K-nearest neighbors (KNNs) were the predominant techniques utilized in this field. These algorithms proved effective in capturing the temporal dependencies and classifying sign gestures based on EMG data during that period. However, as time progressed, between 2014 and 2018, there was a shift towards artificial neural networks (ANNs) and support vector machines (SVMs) for sign language recognition. This transition likely occurred due to the increasing availability of more extensive datasets and advancements in computational resources, enabling the application of more complex models. Lastly, between 2018 and 2023, convolutional neural networks (CNNs) and long short-term memory (LSTM) models emerged as the prominent approaches for EMG-based sign language recognition. The utilization of CNNs and LSTMs indicates a growing interest in leveraging the power of deep learning techniques to extract meaningful features from EMG signals and accurately classify sign gestures. These observations highlight the evolving landscape of sign language recognition techniques over time, driven by advancements in machine learning and increased understanding of EMG signal processing.

### 5.1. K-Nearest Neighbor-Based Approaches

The K-nearest neighbors (KNNs) algorithm is a supervised learning algorithm utilized for classification and regression tasks. Its principle involves identifying the k-nearest data points to a given input and classifying the input based on the majority class among those neighbors. In the domain of EMG signals, KNNs can be employed to classify different types of movements based on the collected EMG data. Sign language recognition systems also utilize KNN-based approaches. For instance, Amor et al. [95] developed a human–machine interface using the Myo armband, which contains eight EMG sensors placed on the forearm. They conducted a study utilizing the MFCC and TDW techniques, along with the KNN classifier to recognize seven hand configurations in French sign language (LSF) in real time. The dataset for this study comprised 2480 samples obtained from 4 healthy subjects, achieving an average accuracy of 90%. KNNs were also applied by Kim, J. et al. [94] for sign language recognition, specifically for recognizing seven words in German sign language (GSL). The authors utilized K-nearest neighbor (kNN) and support vector machine (SVM) algorithms. They collected a dataset of 560 samples from 8 users using 2 EMG sensors and a 3-axis accelerometer attached to the arm. According to the authors, the KNN classifier outperformed the SVM classifier in terms of recognition results, yielding an accuracy of 96.31%. In another study [96], the researchers employed different classifiers, including support vector machines (SVMs), decision tree (DT), linear discriminant analysis (LDA), and K-nearest neighbor (KNN), for recognizing 20 commonly used Persian sign language (PSL) gestures using both EMG and IMU data. The system utilized a low-cost, six-axis IMU (accelerometer and gyroscope), along with a four-channel sEMG and an Arduino as the mainboard for feature extraction. The dataset was collected from 10 subjects, with each subject performing the 20 PSL signs 10 times, resulting in a dataset of 2000 instances. The KNN classifier achieved the highest recognition rate of 96.13% when using 25 selected features. The authors mentioned that while the SVM RBF classifier can provide higher accuracies, it is computationally intensive and complex. Therefore, they found the KNN classifier to be more practical due to its satisfactory accuracies.

### 5.2. Support Vector Machine-Based Approches

In recent times, support vector machines (SVMs) have emerged as a popular choice for processing EMG pattern recognition tasks in sign language recognition using EMG signals. SVMs offer a robust classification method that excels in handling high-dimensional data and achieving high classification accuracy, even with limited training samples. Consequently, SVMs are well-suited for sign language recognition tasks characterized by high-dimensional data and a scarcity of training samples. Researchers have successfully employed SVMs in various sign language recognition systems. For instance, Divya et al. [56] utilized the support vector machine (SVM) to recognize five Indian sign language words obtained from six subjects. The signals representing different signs were captured using BIOPAC-MP-45, and the dataset employed in this study comprised 250 samples. Remarkably, this approach achieved an average accuracy of 90%. To achieve real-time recognition of 80 commonly used American sign language (ASL) words, Jian et al. [36] proposed an innovative approach that combines the inertial measurement unit (IMU) and surface electromyography (sEMG) for detecting hand gestures. They employed four distinct algorithms—decision tree (DT), support vector machine (LibSVM), nearest neighbor (NN), and naïve Bayes—to analyze a dataset comprising 24,000 instances. The data, collected from four subjects, involved utilizing the InvenSense MPU9150, which integrates various sensors like EMG, accelerometer, gyroscope, and magnetometer. Their approach achieved an average accuracy of 96.16%. In another notable study [37], the researchers employed surface electromyography (sEMG) alongside a wrist-worn inertial sensor to classify the forty most frequently used words in American sign language (ASL), gathered from four subjects. They applied a feature selection filter and tested multiple ranking algorithms, including support vector machine (LibSVM), naïve Bayes, decision tree, and nearest neighbor. Their proposed system demonstrated a good average classification rate of 95.94% for the selected set of 40 ASL words.

Cassandra et al. [42] developed a sign language recognition (SLR) system that utilizes surface electromyography (sEMG) signals and an accelerometer. This system is capable of recognizing a set of 50 commonly used American sign language words. The dataset employed in their study was collected from 10 subjects, each wearing two Myo armbands placed on their forearms. To gather an adequate amount of data, each subject performed the different signs 20 times, resulting in a total dataset of 10,000 instances. The proposed approach employed the support vector machine (SVM) algorithm for classification. Three validation techniques were applied to the dataset. Firstly, a 5-fold cross-validation was performed on the training set to assess the model’s performance. Secondly, holdout validation was conducted using data exclusively from the training subjects. Lastly, leave-one-out validation was employed, where one subject’s data was excluded from training the classifier to evaluate its generalizability. The achieved accuracy was 33.66%. In their research, Gupta [57] proposed a machine learning approach based on a radial basis kernel and multiclass support vector machine (SVM) for Indian sign language recognition. They utilized the Delsys Trigno Wireless EMG system, which was placed on the forearm and consisted of one accelerometer sensor and three electromyography (EMG) signal sensors. The study involved 10 hand configurations, and data was collected from 6 subjects, resulting in a dataset of 1200 samples. The authors evaluated the classification accuracy using different data sources. The accuracy achieved using only the accelerometer was 82.14%, while when using only the sEMG signal, it increased to an average accuracy of 86.29%. The best accuracy of 87.5% was attained when both the sEMG and accelerometer data were combined. In a separate study by João et al. [66], supervised machine learning based on binary support vector machines (SVMs) was developed for recognizing 20 letters from Brazilian sign language (LIBRAS). They used the electromyogram (EMG) signals provided by the Myo armband. The signals used in this approach were obtained from a single subject. For each letter, 110 samples were used for training. The accuracy of the collected dataset varied between 4% and 95% depending on the shape of the hand. The authors acknowledged substantial limitations despite being able to identify the gestures based on the obtained results.

Celal and Ferat [38] developed a sign language recognition (SLR) approach using the support vector machine (SVM) and ensemble learning (bagged tree) algorithms. Their system aimed to recognize 27 American sign language (ASL) gestures, which included one gesture for the neutral position and twenty-six gestures representing English alphabet letters. The surface electromyography (EMG) signal was acquired from the Myo armband, placed on the right forearm of 10 subjects. Various feature extraction methods were employed in the study, including power spectral density, time domain, average power, and frequency domain. For time- and frequency-domain feature extraction, the approach utilized FFT band power characteristics and PSD band power characteristics. Through experimentation with both single subject (approximately 2080 samples) and multi-subject (approximately 10,400 samples) datasets, the authors found that the mean power in each channel characteristic yielded the best results among the feature extraction methods. Additionally, they observed that the band power characteristics of FFT outperformed the band power characteristics of PSD. When evaluating the classifiers, the bagged tree and SVM algorithms, the authors noted that the accuracy obtained with the single-subject dataset was superior to that of the multi-subject dataset. Specifically, the bagged tree classifier achieved an accuracy of 80% with the single-subject dataset, while the SVM classifier achieved an accuracy of 60.85%. Consequently, the authors concluded that the bagged tree classifier outperformed the SVM classifier in their SLR system.

### 5.3. Hidden Markov Model-Based Approaches

Hidden Markov models (HMMs) are statistical tools widely used for modeling sequential data, including speech and sign language. They have been employed to recognize patterns in data, such as the transition between sign language gestures. In the context of sign language recognition, HMMs are often combined with electromyography (EMG) signals to analyze muscle movements, gaining insights into hand gestures and enhancing sign language recognition systems. Xu et al. [15] demonstrated the effectiveness of multi-channel EMG and three-axis accelerometer (ACC) sensors for hand gesture classification in Chinese sign language. They utilized multi-stream hidden Markov models and a decision tree to recognize 72 words, achieving accuracies of 95.3% and 96.3% for two subjects. Their continuous sign language recognition system achieved an accuracy of 72.5% for 40 sentence types and an overall word accuracy of 93.1%. In another study by the authors of [16], eight EMG sensors and two three-dimensional accelerometers (3D-ACCs) placed on the right forearm were used. Employing a machine learning technique based on decision trees and multi-stream HMMs, the authors recognized 121 Chinese sign language subwords. The dataset, collected from a single subject, consisted of 2420 instances, and an average accuracy of 95.78% was achieved. Additionally, the study published by the authors of [20] proposed a pattern recognition technique for continuous phonology and component Chinese sign language classification using ACC and sEMG signals. They employed four sEMG sensors and a tri-axial accelerometer, training a machine learning model with a HMM algorithm and dynamic time warping (DTW) technique. Experimental analysis involving 53 base units and 223 characters achieved an accuracy of 96.01% ± 0.83% for the base units and 92.73% ± 1.47% for the characters. The dataset consisted of 36 samples for each character and 20 repetitions for 5 experimental durations, totaling 34,524 samples collected from 5 right-handed subjects. Moreover, Yun Li et al. [27] utilized a multi-stream hidden Markov model (MSHMM) combined with linear discriminant classifiers (LDCs) and Gaussian mixture models (GMMs) for Chinese sign language recognition. They employed a combination of a three-axis accelerometer (ACC) and four surface electromyographic (sEMG) sensors placed around the forearms of five subjects. The dataset contained ACC and EMG data from 200 sentences and 120 signs, with 2400 sentence samples. The system achieved an accuracy of 86.7% for sentences and 96.5% for a vocabulary of 120 signs. In summary, the integration of HMMs with EMG and ACC signals has shown promising results in sign language recognition, with high accuracies achieved in various studies across different datasets and sign languages.

### 5.4. Artificial Neural Network-Based Approaches

In recent years, researchers have found artificial neural network (ANN) tools to be highly promising, particularly in the field of sign language recognition using EMG signals. ANNs offer several advantages, including their ability to handle ambiguous and uncertain data, which is crucial in EMG signal analysis. Moreover, ANNs are renowned for their excellent generalization capabilities. The study published by the authors of [18] presented a system that employed an inertial measurement unit (IMU), gyroscope, and surface electromyography (sEMG) sensor. The Myo armband served as the signal acquisition device, capturing 48 unique words, each repeated 100 times. The study utilized wavelet denoising techniques and applied segmentation using the Teager–Kaiser energy operator (TKEO) thresholds. They explored the use of a feature-based artificial neural network (ANN) to recognize 48 words in Chinese sign language, achieving an impressive success rate of 97.12%. Varadach et al. [98] utilized the “TMS porti” system, which was placed around the forearm muscles for recording EMG signals. This system featured eight-channel electrodes and served as the data acquisition tool. To recognize 10 Thai sign language alphabet letters, the authors proposed an approach based on ANN artificial neural networks using the backpropagation technique. The dataset employed in this study comprised a total of 2000 samples collected from a single subject, with each alphabet repeated 200 times. The experimental analysis of the dataset yielded an average accuracy of 95%.

In the study published by the authors of [86], a wireless armband featuring eight sEMG sensors was developed and designed for recording sEMG signals at a frequency of 600 Hz. The armband was placed on the right forearm and used to capture the sEMG signals associated with 38 hand shapes in Korean sign language (KSL), including 14 consonants, 17 vowels, and 7 digits. The researchers proposed an approach based on an E-ANN structure, which involved combining multiple ANN classifiers. This approach achieved an impressive accuracy of 97.4%. During testing, the authors determined that the optimal configuration for this approach involved eight ANN classifiers and a sample size of three hundred. Another approach, introduced by Cristina et al. [40], utilized a simple neural network architecture consisting solely of fully connected layers and employing the leaky ReLU activation function. The researchers employed a Myo armband to record EMG signals from thirteen hand shapes, including eight hand gestures and five letters (‘V’, ‘L’, ‘I’, ‘F’, and ‘D’), in American sign language (ASL). The dataset used in this approach comprised 1300 samples collected from 50 subjects, with each gesture repeated 2 times. The system achieved an accuracy of 99.78% for the recognition of 7 gestures and 99.31% for the recognition of all 13 gestures.

In the study published by the authors of [41], the researchers employed the principal component analysis (PCA) filter, along with a multilayer perceptron neural network (MPN), for the classification of surface EMG signals associated with nine words from American sign language (ASL). The experiments were conducted using the SCEPTRE database, which consisted of data captured from two Myo armbands placed on the forearms of two hands belonging to three subjects. The dataset used in these experiments included several attributes, such as 6 gyroscope vectors, 6 accelerometer vectors, 6 position vectors, and 16 EMG vectors, resulting in a range from 34 to 20 attributes. The average precision for the recognition of the nine words by the three subjects was as follows: When using the attributes sEMG, accelerometer, gyroscope, and orientation (34 attributes), the precision obtained was 100% with the multilayer perceptron and 97.77% with the multilayer perceptron using PCA. With the sEMG, accelerometer, and gyroscope attributes (29 attributes), the accuracy obtained was 99.25% with the multilayer perceptron and 94.81% with the multilayer perceptron using PCA. Using the sEMG, accelerometer, and orientation attributes (29 attributes), the accuracy obtained was 100% with the multilayer perceptron and 99.25% with the multilayer perceptron using PCA. By utilizing the sEMG and accelerometer attributes (23 attributes), the accuracy achieved was 95.55% with the multilayer perceptron and 90.62% with the multilayer perceptron using PCA. When using the sEMG and gyroscope attributes (23 attributes), the accuracy obtained was 77% with the multilayer perceptron and 74.07% with the multilayer perceptron using PCA. With the sEMG and orientation attributes (23 attributes), the accuracy obtained was 93.44% with the multilayer perceptron and 93.33% with the multilayer perceptron using PCA. Finally, using only the sEMG attributes (16 attributes), the accuracy achieved was 47.4% with both the multilayer perceptron and the multilayer perceptron using PCA.

In another study by A.L.P Madushanka et al. [99], a system was developed based on nine different artificial neural network (ANN) classifiers. The system utilized various sensors of the Myo armband, including the inertial measurement unit (IMU) sensors (accelerometer, gyroscope, and magnetometer/orientation) and the surface electromyography (sEMG) sensor, to recognize 12 signs in Sinhala sign language. The study involved six subjects, with each subject repeating the twelve signs five times. The dataset used in this study comprised a total of 360 samples, and the accuracy achieved was 94.4%. In the study published by the authors of [24], a real-time Chinese sign language (CSL) recognition model was proposed for the classification of 15 CSL gestures extracted from sEMG signals acquired by the Myo armband. The study involved 10 healthy subjects, and a total of 5250 samples were utilized. Several techniques were employed for EMG signal processing and feature extraction, including the energy spectrum approach, short-term Fourier transform, sliding window approach, and t-distributed stochastic neighbor embedding (T-SNE). An ANN algorithm with three layers was trained using machine learning for this application. Experimental results demonstrated an accuracy of 88.7% for the recognition of the 15 signs.

### 5.5. Convolutional Neural Network-Based Approaches

Convolutional neural networks (CNNs) are a specific type of neural network that excel in image classification tasks. They have been widely employed in several applications, such as object detection, image recognition, and facial expression recognition. In the domain of electromyography (EMG), CNNs have also been utilized for hand gesture recognition in sign language. For instance, in the work presented by the authors of [14], a real-time system was developed using deep learning to recognize 86 words in Chinese sign language. The dataset consisted of data collected from 20 subjects using the surface electromyography (sEMG) and inertial measurement unit (IMU) sensors of the Myo armband. The proposed approach utilized the CNN algorithm based on the VGG architecture. It achieved an average precision ranging from 94.72% to 98.92%. In another study by Qian et al. [39], a deep learning-based system named MyoSign was proposed for American sign language (ASL) recognition. The system leveraged both inertial and EMG signals. The authors introduced a multimodal approach that combined convolutional neural networks (CNNs), long short-term bidirectional memory (LSTM), and connectionist temporal classification (CTC). The system achieved a word precision of 93.7% and a sentence accuracy of 93.1% when matching 70 commonly used ASL words and 100 ASL sentences from 15 subjects, utilizing a lightweight handheld device capable of capturing EMG and inertial signals. In the study published by the authors of [84], the researchers employed a scale average wavelet transform and a basic CNN classifier to recognize three manual signs and three handshape sign language gestures. The EMG signals were obtained from a single subject using the Myo armband, resulting in a dataset of 1200 samples. The average accuracy achieved using this approach was 94%. Furthermore, in the study published by the authors of [85], a recognition system was developed using both CNNs and LSTMs to recognize 30 Korean sign language gestures. The EMG and IMU sensors of the Myo armband were utilized to capture the signals corresponding to different signs. Initially, the learning architecture of the neural network was created using data from a single subject, and subsequently, an accuracy of 99.6% was achieved. Overall, CNNs have proven to be effective in sign language recognition tasks, demonstrating high accuracy rates in various studies across different sign languages and datasets.

### 5.6. Long Short-Term Memory-Based Approaches

Long short-term memory (LSTM) is a type of recurrent neural network (RNN) known for its ability to capture long-term dependencies in sequential data. Within the electromyography (EMG) signal context, LSTMs have been employed to analyze and classify various movement patterns. Researchers have successfully utilized LSTMs to classify hand and finger movements in EMG signals, demonstrating their potential in EMG signal analysis and sign language recognition systems. For instance, Paolo S et al. [81] combined surface IMU and EMG data obtained from the Myo gesture control armband to develop a deep neural network based on bidirectional LSTM architecture for recognizing 26 letters of Italian sign language (LIS). The dataset consisted of 30 samples for each letter, totaling 780 samples collected from a single individual wearing a Myo armband on the right arm. The average accuracy achieved using this approach was 97%. LSTMs were also utilized in the research by the authors of [78], where CNNs (convolutional neural networks) and LSTMs were combined for recognizing 28 hand shapes representing Arabic alphabet letters. Electromyographic signals from the eight sensors of the Myoware armband were used, and 400 samples were recorded for each hand shape. In total, three subjects contributed to the recording of a dataset of 9350 samples. The approach involved seven CNN layers, an LSTM layer, and a GRU layer for feature extraction, followed by two dense neural network layers for classifying the twenty-eight letters in ArSL. The average precision obtained in this study was 98.49%.

In another study published by the authors of [80], a deep learning model composed of two sub-networks was developed to recognize seven hand gestures in Arabic sign language (ArSL). The EMG signals were obtained from the eight sensors of the Myoware armband, which were evenly placed around the forearm. The dataset, involving 8 subjects (4 deaf and 4 hearing), comprised a total of 15,000 samples. The authors observed that increasing the number of sensors improved the accuracy of the results. Therefore, they proposed acquiring the data from the eight Myoware armband sensors and estimating the signal curve using barycentric interpolation. The proposed deep learning approach utilized CNNs with expanded convolution for feature extraction from EMG signals and two LSTM layers for extracting temporal features from sEMG signals. The recognition rate achieved using this approach was 97.4%. Moreover, a study by the authors of [43] achieved an accuracy of 97.9% for 20 signs performed by 20 subjects, utilizing sEMG signals obtained from the Myo armband placed on the subjects’ forearms. The researchers developed a machine learning model based on the bilinear model and LSTM, with 10 samples recorded for each sign. The dataset used in this study consisted of 4000 instances.

### 5.7. Other Proposed Approaches

Jinuk K et al. [70] proposed an approach for sign recognition that extracted sign language primitives using motion sensors and surface electromyography (EMG) data obtained from eight EMG sensors and three-axis IMU sensors of the Myo armband. In this approach, 12 different hand shapes were classified as a handshape vector by summing the readings from different Myo armband sensors placed at the brachial level. Additionally, six hand motion primitives were classified as motion direction vectors by calculating Euler’s angle differences. The authors claimed that their proposed primitive sign language extraction system could quickly and accurately identify sign language words, thereby reducing the computational load in sign language recognition. In another study, Muhammad U et al. [87] proposed a supervised machine learning approach based on a linear discriminant classifier to recognize the 26 alphabet letters of Pakistani sign language. They employed empirical mode decomposition (EMD) to remove unwanted interferences from the signal. Three sEMG sensors connected with the set SS2LB were placed on the interior side of the forearm to acquire the sEMG signals. The BIOPAC system was used to collect and convert the analog signals into digital signals. Each alphabet was repeated 30 times, resulting in a dataset of 780 samples. The success rate achieved using this technique was 81%.

In a study by the authors of [12], an approach was developed to recognize the 26 alphabet letters of Irish sign language using sEMG data obtained from the eight sensors of the Myo armband. Initially, only sEMG data were used, but later IMU data were added, resulting in a 10% increase in accuracy. The dataset used in the study was collected from 12 subjects wearing Myo armbands on both forearms. Each subject performed the different signs five times, resulting in a total of 1560 instances. To improve the results, the authors customized their feature selection approach using the “feature importance” function of the random forest classifier. This helped identify the most important features for letter recognition and adjust their methods accordingly. The study employed various machine learning techniques from the scikit-learn toolkit, including linear regression, naïve Bayes, random forest, ensemble methods, and support vector machines. Through experimentation and optimization, they achieved an accuracy of 78%, with the best accuracy obtained using models based on the random forest and ensemble methods. In another study conducted by Yi et al. [17], the deep learning method was applied for Chinese sign language recognition based on wearable sensors. The proposed approach utilized a deep belief net (DBN) and involved the combination of three different sensors: electromyography (sEMG), accelerometer (ACC), and gyroscope (GYRO). The study focused on recognizing 150 Chinese sign language subwords obtained from 8 subjects. Each subject repeated the subwords 5 times in each of 5 sessions, resulting in a total of 3750 CSL subwords collected per subject. Three sensor fusion strategies, namely data-level fusion, feature-level fusion, and decision-level fusion, were explored. The approach achieved an average accuracy of 95.1% for user-dependent testing and 88.2% for user-independent testing.

In a study by Simin Yuan et al. [21], sEMG signals were utilized to recognize 30 Chinese sign language alphabet letters. The Delsys Trigno division, which contains eight sEMG sensors placed on the forearm, was employed for data collection from four hearing subjects. The dataset used in this study comprised 600 samples. To enhance the accuracy of the machine learning model, the researchers first applied a notch filter to preprocess the raw data. They then utilized several techniques for feature extraction, including mean absolute value (MAV), fourth-order autoregressive (AR) coefficients, Wilson amplitude (WAMP), and waveform length (WL). The proposed unsupervised learning method employed the random forest algorithm, which combines various unique classifiers through bootstrapping to build decision trees for signal analysis and decision making. The authors claimed that the random forest algorithm outperformed artificial neural networks (ANNs) and support vector machines (SVMs) for most gestures, although misclassifications still occurred for similar letters of the alphabet. The approach achieved an average accuracy of 95.48%. In another study by Yongjie Z et al. [23], an approach was proposed for recognizing 18 isolated Chinese sign language (CSL) signs. The approach was based on features extracted from both accelerometer (ACC) and surface electromyography (sEMG) data. The linear discriminant analysis (LDA) classifier was employed, and the dataset used consisted of 864 instances. Data was collected from eight subjects using three sEMG sensors and two accelerometers from the DELSYS TrignoTM Wireless EMG System. The authors obtained an average accuracy ranging from 84.9% to 91.4% using this approach. They noted that placing the sEMG and ACC sensors on the back of the hand improved the recognition rate by an additional 6.5%, resulting in an accuracy of 91.4%. In contrast, an accuracy of 84.9% was achieved when the sensors were only placed on the forearm and wrist.

In a study conducted by Ruiliang et al. [25], it was demonstrated that multi-channel electromyography (EMG) and three-axis accelerometers can be utilized for hand gesture recognition. The researchers developed an acquisition device specifically for this purpose and collected data for 121 Chinese sign language (CSL) subwords. In their setup, four sEMG sensors and a three-axis accelerometer were positioned around the left and right forearms. One sEMG sensor and the three-axis accelerometers were placed near the back of the wrist, while the other three sEMG sensors were placed near the elbow on the forearm. The dataset used for experimental analysis consisted of 12 instances, with 12 samples for each subword obtained from 5 subjects. The approach employed the random forest algorithm as the primary classification framework. It consisted of a pre-classifier, a hand-orientation classifier, two-handed classifiers, one-handed classifiers, and a multi-stream hidden Markov model (HMM) classifier. To ensure improved accuracy of the random forests, an enhanced decision tree was used. The approach achieved an accuracy of 98.25%. The authors acknowledged two main limitations of their approach. Firstly, the construction time of the random forest with all decision trees was found to be excessively long. Secondly, the sensor placement could have potentially influenced the performance of the proposed sign language recognition (SLR) system.

## 6. Discussion

It is true that many sign language recognition systems based on electromyography (EMG) signals have shown promising feasibility due to significant advancements in their design. These systems utilize EMG data alone or in combination with inertial measurement unit (IMU) data to improve gesture recognition accuracy in sign language. However, the use of sEMG signals also presents challenges, as they are susceptible to various factors that can impact the reliability and performance of sign language recognition systems. One of the highlighted challenges is the variability of sEMG data from person to person and even from hour to hour, as it depends on factors such as the muscular state, including the levels of strength and fatigue. This variability makes it important to consider individual differences when designing and deploying these systems. The location of the sensors on the forearm is another crucial factor to consider. The size of the forearm, particularly for approaches that utilize devices like the Myo armband, can affect the positioning and effectiveness of the sEMG sensors. Additionally, the physiological characteristics of muscles differ from person to person, which further emphasizes the need for individualized placement of the sEMG sensors on the forearm muscles. Moreover, the use of multiple sensors has been found to enhance the reliability of sign language recognition systems. By incorporating data from different sensors, the system can capture a more comprehensive representation of the user’s gestures, improving overall accuracy.

**Figure 11:**
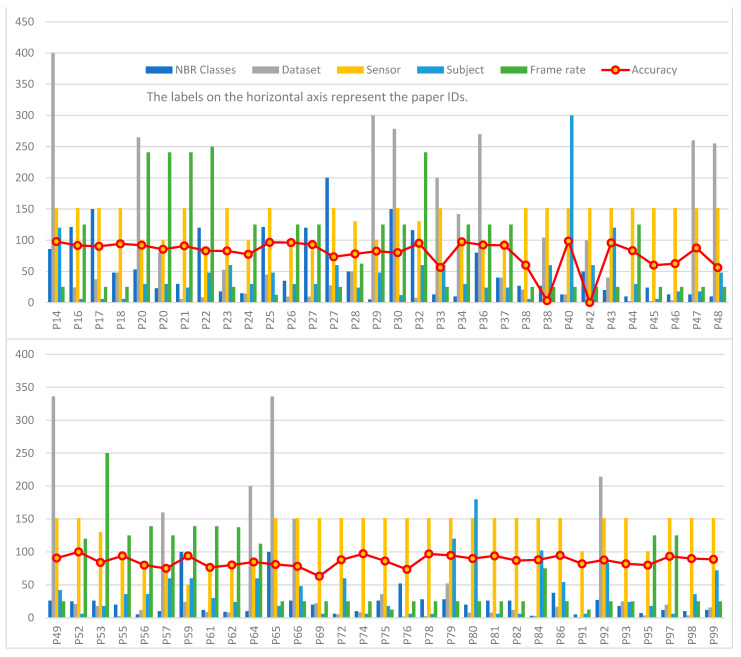
Comparative analysis of accuracy in the selected papers relative to dataset size, number of classes, number of sEMG channels, and number of subjects.

In Figure 11, we present an analysis illustrating the relationship between accuracy and various parameters, including data size, number of classes, number of sEMG channels, and number of subjects in the selected papers. Examining the number of subjects allowed us to identify three papers with a larger participant pool: The studies published by the authors of [31,40,81]. The achieved accuracy in these papers exceeds 97%, with the exception of the study published by the authors of [31], where the accuracy has not been defined. Notably, the study published by the authors of [81] achieved an accuracy of 97% in recognizing 26 gestures, performed by 30 subjects, utilizing a reasonably sized dataset. This finding is noteworthy, given the known instability of sEMG signals from one individual to another.

To address the recognition challenges, several approaches discussed in these research papers have employed machine learning techniques with various algorithms, segmentation methods, and feature extraction techniques. These machine learning-based approaches leverage the power of algorithms to learn from the sEMG data and improve the recognition accuracy. Deep learning, in particular, has been employed in some approaches due to its ability to effectively learn from sEMG data, reducing the heterogeneity of data discrepancies between different types of sensors. This, in turn, leads to a better performance of the sign language recognition systems. Based on the provided information, the survey focused on various common sign language recognition approaches using surface electromyography (sEMG) signals. The classifiers used in these approaches include support vector machines (SVMs), artificial neural networks (ANNs), convolutional neural networks (CNNs), K-nearest neighbors (KNNs), hidden Markov model (HMMs), and long short-term memory (LSTM). The survey highlighted that SVMs and ANNs are the most commonly used classifiers for gesture recognition in sign language using EMG signals. SVM classifiers, particularly the LibSVM variant, have been improved through incorporating the decision tree, nearest neighbor, and naïve Bayes methods to enhance accuracy in certain studies. However, it was mentioned that ANN and SVM classifiers may not always provide the best precision. The article published by the authors of [21] claims that the random forest algorithm outperforms the ANN and SVM classifiers in classifying most of the gestures. Additionally, research conducted by the authors of [94] demonstrated that the KNN classifier achieves better recognition results compared to the SVMs. However, the KNN classifier is computationally intensive and complex. In the study published by the authors of [96], the KNN classifier was found to offer more satisfactory precision than SVMs, although SVMs provide higher accuracies. Regarding ANN models, various types have been utilized, including the emotional artificial neural network (E-ANN), multilayer perceptron, and backpropagation neural network. The research conducted by the authors of [41] reports the highest accuracy of 100% using a multilayer perceptron neural network classifier with data from the Myo armbands. CNNs have been identified as the third most popular classifier for sign language gesture classification using EMG signals. Several studies combine CNNs with LSTMs to improve accuracy, as seen in approaches published by the authors of [78] and [80]. The highest accuracy achieved among these approaches was 99.6% [85], utilizing both EMG and inertial measurement unit (IMU) data. Among the learning algorithms mentioned, LSTMs and CNNs have shown promising results in sign language gesture classification using sEMG data. However, LSTM is generally considered the best algorithm due to its suitability for sequential data and ability to capture temporal dependencies. LSTM networks can retain information from previous inputs over longer periods, allowing them to better handle sequential dependencies in EMG signals, which is crucial for sign language recognition. In summary, the survey highlights the prevalence of SVM and ANN classifiers in sign language recognition using EMG signals. While some studies suggest alternative classifiers, such as the random forest and KNN classifiers, LSTM stands out as the most effective algorithm for capturing sequential dependencies and improving gesture recognition performance in EMG-based sign language recognition systems.

In conclusion, sign language recognition systems based on sEMG signals have made significant progress thanks to advancements in their design. While challenges related to variability in sEMG data and sensor placement exist, machine learning and deep learning techniques have been employed to tackle these challenges and improve the accuracy and reliability of these systems. The use of multiple sensors has also been found beneficial in achieving better performance.

## 7. Conclusions

Our article represents a systematic review of the literature of studies on the recognition of sign languages published in the last decade. A total of 88 papers related to sign language recognition represent our dataset which were analyzed; they were collected from different academic databases, namely Google Scholar, Scopus, Web of science, Springer Link, IEEE Xplore, and ACM Digital Library. This review demonstrates that sign language analysis and recognition, which recognizes signs using EMG signals, is a very recent and emerging area of research. Most of the studies reviewed use both sEMG and IMU data, while a relatively limited number of studies only use sEMG data for sign language gesture recognition. We distinguish in this article several approaches that differ by the method of analysis, the method of learning, the classifier, the devices for acquiring signs, as well as the dataset that includes the number of gestures and the number of subjects; all these parameters influence the performance of the sign language recognition systems proposed in the various studies examined. Thus, these approaches showed promising results. On the other hand, it is difficult to make a reliable comparison of these approaches, as the studies that used a reference database were very limited; however, most of the studies used their own dataset, which were collected by themselves. In this context, it is preferable to use calibrated databases in future work so that the comparison between the different approaches is more concrete.

## Figures and Tables

**Table 1:** Comparative overview of technologies for sign language recognition.

Parameter	Video	Motion Capture	Surface Electromyography
Sensing technology	Cameras	Infrared cameras/sensors	Electrodes
Data type	Visual/2D/3D	3D positions/orientations	Muscle activation signals
Sensitivity	Light conditions	Marker occlusions	Muscle contractions and noise
Gesture types	Static/dynamic	Static	Static/dynamic
Spatial resolution	High (depends on camera)	High	Moderate/high
Temporal resolution	High (depends on fps)	High	High
Accuracy	Variable (depends on algo)	High (depends on setup)	Variable (depends on algo and setup)
Application	General sign language	Detailed motion analysis	Muscle analysis for sign language
Portability	Moderate/high	Low	High
Cost	Low/moderate	High	Moderate

**Table 2:** Papers on sign languages.

Sign Language	Percent	References
Chinese sign language	23.86%	[14,15,16,17,18,19,20,21,22,23,24,25,26,27,28,29,30,31,32,33,34]
American sign language	23.86%	[35,36,37,38,39,40,41,42,43,44,45,46,47,48,49,50,51,52,53,54,55]
Indian sign language	11.4%	[56,57,58,59,60,61,62,63,64,65]
Brazilian sign language	4.5%	[66,67,68,69]
General sign language	4.5%	[70,71,72,73]
Indonesian sign language	4.5%	[74,75,76,77]
Arabic sign language	3.4%	[78,79,80]
Italian sign language	3.4%	[81,82,83]
Korean sign language	3.4%	[84,85,86]
Pakistani sign language	2.3%	[87,88]
Turkish sign language	2.3%	[89,90]
Malaysian sign language	1.1%	[91]
Peru sign language	1.1%	[92]
Polish sign language	1.1%	[93]
German sign language	1.1%	[94]
French sign language	1.1%	[95]
Parisian sign language	1.1%	[96]
Colombian sign language	1.1%	[97]
Thai sign language	1.1%	[98]
Sinhala sign language	1.1%	[99]
Irish sign language	1.1%	[100]
Greek sign language	1.1%	[101]

**Table 3:** sEMG datasets for sign language recognition.

Sign Language	Nbr Classes	Type	Subjects	Size	Device
Arabic sign language	28	Alphabet	3	9350	Myo armband
Italian sign language	26	Alphabet	1	780	Myo armband
American sign language	10	Word	8	320	Myo armband
Indian sign language	6	Sentence	19	223	Myo armband
American sign language	26	Alphabet	9	234 × 5 s	Myo armband
General sign language	5	Emotion	12	360	-

**Table 4:** Features used for EMG-based SL recognition.

Feature	Paper Count	Feature Class	Paper Count
Variance (VAR)	17	Time-domain/statistical features	138
Mean absolute value (MAV)	46		
Modified mean absolute value	4		
Root mean square (RMS)	27		
Standard deviation (SDV)	20		
Average amplitude change (AAC)	8		
Maximum (MAX)	6		
Minimum	4		
Median	4		
Average power	2		
Modified mean frequency (MMF)	3	Frequency-domain features	30
Mean frequency (MFR)	7		
Modified median frequency	2		
Median frequency	5		
Reflection coefficient	1		
Power spectral density	2		
Discrete Fourier transform	2		
Spectral mean	1		
Spectral standard deviation	1		
Spectral skewness	1		
Maximum energy frequency	1		
Power in the channel	2		
Standard deviation (SDV)	2		
Temporal and spectral moment	2	Time-frequency features	6
Moving variance	2		
Short-time Fourier transform	2		
Histogram	2	Signal shape and distribution Features	8
Minimum fractal length	1		
Maximum fractal length	4		
Shape factor	1		
Kurtosis (KUR)	9	Higher-order statistics	20
Skewness (SKW)	11		
Mel frequency cepstral coefficient	3	Mel frequency cepstral coefficients	4
Mean of gammatone cepstral coefficient	1		
Wavelet transform	3	Wavelet transform coefficients	5
Scale-average wavelet transform (SAWT)	1		
Wavelet energy	1		
Autoregressive coefficient (ARC)	13	Autoregressive model coefficients	13
Waveform length (WVL)	21	Waveform-based features	49
Zero crossing rate (ZCR)	17		
Willison amplitude	4		
Simple square integral (SSI)	7		
Log detector (LGD)	5	Other features	37
Sample entropy	1		
Permutation entropy	1		
Mean power	1		
Power spectrum ratio	1		
Peak frequency	2		
Spurious-free dynamic range	1		
Log energy	1		
Shannon energy	2		
Irregularity factor	1		
Katz fractal dimension	1		
Integrated absolute value	3		
Slope sign changes	11		
Hjorth parameter	2		
Linear prediction coefficient	1		
Difference absolute standard deviation value	2		
Root squared zero-order moment normalized	1		

## Data Availability

Not applicable.

## Appendix A

**Table A1:** Used hardware.

Ref.	Sensor	Device	EMG Channel	Freq.	Hand
[14]	sEMG + accelerometer + gyroscope + magnetometer	Myo armband	8	200 Hz	Right
[15]	sEMG + accelerometer	-	5	1000 Hz	Right
[16]	sEMG + 2 accelerometers	-	8	1000 Hz	Right/tow
[17]	sEMG + 2 accelerometers + 2 gyroscopes	-	8	1000 Hz	Right
[18]	sEMG + accelerometer + gyroscope	Myo armband	8	200 Hz	-
[19]	sEMG	Custom device: Conductor muscle electrical sensor, Arduino UNO.	6	-	-
[20]	sEMG + accelerometer	Delsys Trigno Lab Wireless System	4	1927 Hz	Right
[21]	sEMG	Delsys Trigno	8	1926 Hz	Right
[22]	sEMG + accelerometer	Custom device	4	1 kHz	Tow
[23]	sEMG and accelerometer	DELSYS TrignoTM Wireless EMG System	3	2000 Hz	Right
[24]	sEMG	Myo armband	8	200 Hz	-
[25]	sEMG + accelerometer	Custom device: sEMG sensors, MMA7361	4	1000 Hz	Tow
[26]	sEMG + accelerometer + gyroscope + magnetometer	Myo armband	8	100 Hz	-
[27]	sEMG + 2 accelerometers	Custom device: NIPCI-6036 E, National Instruments	8	1000 Hz	Tow
[28]	sEMG + accelerometer + gyroscope	Myo armband	8	200 Hz	Right
[29]	sEMG + accelerometer + gyroscope	Custom device	6	500 Hz	Right
[30]	sEMG + accelerometer + gyroscope	Custom device	4	1 kHz	Tow
[31]	sEMG + accelerometer + gyroscope	Myo armband	8	200 Hz	Tow
[32]	sEMG + accelerometer	Custom device	8	1 kHz	Tow
[33]	sEMG	Delsys Trigno Lab Wireless System	6	2 kHz	-
[34]	sEMG + accelerometer + gyroscope + magnetometer	Myo armband	8	200 Hz	Right
[35]	sEMG +acceleration, gyroscope, gravity sensors	Smartwatch + Myo armband	8	200 Hz	Dominant
[36]	sEMG + accelerometer + gyroscope + magnetometer	Custom device: InvenSense MPU9150 ADS1299	4	1000 Hz	Right
[37]	sEMG + accelerometer + gyroscope + magnetometer	Custom device: InvenSense MPU9150, TI ADS1299	4	1000 Hz	Right
[38]	sEMG	Myo armband	8	200 Hz	Dominant
[39]	sEMG + accelerometer + gyroscope + magnetometer	Myo armband	8	200 Hz	Right/tow
[40]	sEMG	Myo armband	8	200 Hz	-
[41]	sEMG + accelerometer + gyroscope + magnetometer	2 Myo armbands	8 each armband	200 Hz	Tow
	sEMG + accelerometer + gyroscope				
	sEMG + accelerometer + magnetometer				
	sEMG + accelerometer + magnetometer				
	sEMG + accelerometer				
	sEMG + gyroscope + magnetometer				
	sEMG				
[42]	sEMG + accelerometer	2 Myo armbands	8	200 Hz	Tow
[43]	sEMG	Myo armband	8	200 Hz	Right
[44]	sEMG + FMG	Custom device: ADS1299, Texas instrument	8	1000 Hz	-
[45]	sEMG + accelerometer + gyroscope + magnetometer	Myo armband	8	200 Hz	-
[46]	sEMG + accelerometer + gyroscope + magnetometer	Myo armband	8	200 Hz	Tow
[47]	sEMG + accelerometer + gyroscope	Myo armband	8	200	Tow
[48]	HD- sEMG	Custom device	8 × 16	400 Hz	Right
[49]	sEMG + accelerometer + gyroscope + magnetometer	Myo armband	8	200 Hz	-
[50]	sEMG + accelerometer + flex	Custom device	2	-	-
[51]	sEMG	Myo armband	8	200 Hz	-
[52]	sEMG + accelerometer + gyroscope + magnetometer + leap motion + VIVE HMD	Myo armband	8	200 Hz	-
[53]	sEMG	Bio Radio 150 CleveMed	8	960 Hz	Right
[54]	sEMG + accelerometer + gyroscope	Myo armband	8	200 Hz	Tow
[55]	sEMG	Delsys Trigno Lab Wireless System	6	2 kHz	Tow
[56]	sEMG	BIOPAC-MP-45	4	1000 Hz	Right
[57]	sEMG	Delsys Trigno Wireless EMG	3	1111 Hz	Dominant
	Accelerometer + sEMG				
[58]	sEMG + accelerometer + gyroscope + magnetometer	**-**	3	1000 Hz	Tow
[59]	sEMG + accelerometer + gyroscope	Delsys Trigno Lab Wireless System	3	1 kHz	Tow
[60]	sEMG	Delsys Trigno Lab Wireless System	5	1 kHz	-
[61]	sEMG + accelerometer + gyroscope	Delsys Trigno Lab Wireless System	2	1 kHz	Dominant
[62]	sEMG	Delsys Trigno Lab Wireless System	3	1 kHz	Dominant
[63]	sEMG	Custom device	1	1 kHz	Right
[64]	sEMG	Delsys Trigno Lab Wireless System	3	1.1 kHz	Right
[65]	sEMG + accelerometer + gyroscope	Delsys Trigno Lab Wireless System	3	900 kHz	Tow
[66]	sEMG	Myo armband	8	200 Hz	-
[67]	sEMG + accelerometer + gyroscope + magnetometer	Myo armband	8	200 Hz	-
[68]	sEMG + accelerometer + gyroscope	Myo armband	8	200 Hz	Right
[69]	sEMG	Myo armband	8	200 Hz	-
[70]	sEMG + accelerometer + gyroscope + magnetometer	Myo armband	8	200 Hz	-
[71]	sEMG	Custom device	4	-	-
[72]	sEMG	Myo armband	8	100 Hz	-
[73]	sEMG + pressure	Custom device	3	-	-
[74]	Leap motion + sEMG	Myo armband	8	200 Hz	Tow
[75]	sEMG + accelerometer + gyroscope + magnetometer	Myo armband	8	200 Hz	-
[76]	sEMG + accelerometer + gyroscope + magnetometer	Myo armband	8	200 Hz	-
[77]	sEMG + accelerometer + gyroscope + magnetometer	Myo armband	8	200 Hz	Tow
[78]	sEMG	Myo armband	8	200 Hz	Right
[79]	sEMG	Custom device	3	500 Hz	Right
[80]	sEMG	Myo armband	8	200 Hz	Right
[81]	sEMG + accelerometer + gyroscope + magnetometer	Myo armband	8	200 Hz	Right
[82]	sEMG	Myo armband	8	200 Hz	-
[83]	sEMG	Myo armband	8	200 Hz	-
[84]	sEMG	Myo armband	8	200 Hz	Dominant
[85]	sEMG + accelerometer + gyroscope + magnetometer	Myo armband	8	200 Hz	Right
[86]	sEMG	sEMG armband	8	600 Hz	Right
[87]	sEMG	BIOPAC	3		Dominant
[88]	sEMG	BIOPAC	3	-	-
[89]	sEMG	Myo armband	8	200 Hz	-
[90]	sEMG + accelerometer + gyroscope + magnetometer	Myo armband	8	200 Hz	-
[91]	sEMG + accelerometer + gyroscope + force-sensing resistor	Custom device	1	10 Hz	Right
[92]	sEMG	Custom device	4	100 Hz	Left
[93]	sEMG + accelerometer + gyroscope + magnetometer	Myo armband	8	200 Hz	Dominant
[94]	sEMG + accelerometer	Custom device	1	-	-
[95]	EMG	Myo armband	8	200 Hz	Right
[96]	sEMG + accelerometer + gyroscope	Custom device	4	-	Right
[97]	sEMG + accelerometer	BTS FREEMG	4	1000 Hz	Right
[98]	sEMG	TMS porti	8	1000 Hz	-
[99]	sEMG + accelerometer + gyroscope + magnetometer	Myo armband	8	200 Hz	Right
[100]	sEMG + accelerometer + gyroscope + magnetometer	Myo armband	8	200 Hz	-
[101]	sEMG + accelerometer	Bioplux8	5	1000 Hz	Right

**Table A2:** Data acquisition main parameters.

Ref.	Target	Type	Classes	Dataset Size	Sensor	Placement	Frame Rate	Subjects	Accuracy
[14]	Chinese SL	Word	86	85,424	8	Armband placed on arm muscles: Extensor carpi radialis longus Flexor carpi ulnaris Flexor carpi radialis Brachioradialis Extensor digitorum Extensor digiti minimi	200	20	From 94.72% to 98.92%
[15]	Chinese SL	Word	72	ni	5	Extensor digiti minimi Palmaris longus Extensor carpi ulnaris Extensor carpi radialis Brachioradialis	1000	2	93.1%
[16]	Chinese SL	Subword	121	2420	8	Extensor digiti minimi Palmaris Extensor carpi ulnaris Extensor carpi radialis	1000	1	95.78%
[17]	Chinese SL	Subword	150	3750	8	Extensor digiti minimi Palmaris longus Extensor carpi ulnaris Extensor carpi radialis	1000	8	From 88.2% to 95.1%
[18]	Chinese SL	Word	48	4800	8	Armband placed on arm muscles: Same as the study published by the authors of [14]	200	1	97.12%
[19]	Chinese SL	Alphabet	4	800	6	Pronator quadratus Flexor digitorum superficialis Flexor carpi ulnaris Palmaris longus Flexor carpi radialis Brachioradialis Pronator teres	-	4	86%
[20]	Chinese SL	Word	53	26,500	4	Extensor digitorum Palmaris longus Extensor carpi radialis longus Flexor carpi ulnari The hybrid sensor (EMG+IMU) is placed on the extensor digiti minimi. Extensor pollicis longus Extensor pollicis brevis	1927	5	96.01 ± 0.83%
		Alphabet	23	8028					92.73% ± 1.47
[21]	Chinese SL	Alphabet	30	600	8	Extensor carpi radialis brevis Extensor digitorum Brachioradialis Extensor carpi ulnaris	1926	4	95.48%
[22]	Chinese SL	Subword	120	14,200	4	-	1000	5	91.51%
[23]	Chinese SL	Word	18	864	3	Extensor carpi radialis longus Extensor carpi ulnaris Flexor carpi radialis longus Extensor digitorum Tendons of extensor digitorum/lumbricals	2000	8	From 84.9% to 91.4%
[24]	Chinese SL	Word	15	5250	8	Armband placed on arm muscles: Same as the study published by the authors of [14]	200	10	88.7%
[25]	Chinese SL	Subword	121	1452	4	Extensor minimi digiti Palmaris longus Extensor carpi ulnaris Extensor carpi radilis	1000	5	98.25%
[26]	Chinese SL	Word	35	4480	8	Armband placed on arm muscles: Same as the study published by the authors of [14]	100	8	98.12%
[27]	Chinese SL	Word Sentence	120	975	4	Extensor minimi digiti Palmaris longus Extensor carpi ulnaris Extensor carpi radialis	1000	5	96.5%
			200						86.7%
[28]	Chinese SL	Word	50	2780	8	Armband placed on arm muscles: Same as [14]	200	10	89%
[29]	Chinese SL	Word	5	5000	6	Extensor digitorum Flexor carpi radialis longus Extensor carpi radialis longus Extensor carpi ulnaris	500	4	91.2%
[30]	Chinese SL	Word	150	30,000	4	Extensor digiti minimi Palmaris longus Extensor carpi ulnaris Extensor carpi radialis	1000	8	90%
[31]	Chinese SL	Word	60	20,400	8	Armband placed on arm muscles: Same as the study published by the authors of [14]	200	34	WER 19.7
[32]	Chinese SL	Subword	116	27,840	8	Extensor minimi digiti Palmaris longus Extensor carpi ulnaris Extensor carpi radialis	1000	2	97.55%
[33]	Chinese SL	Hand shape	13	780	6	Extensor carpi radialis longus Extensor digitorum and flexor carpi ulnaris Palmaris longus Extensor pollicis longus Abductor pollicis longus Extensor digiti minimi	1927	10	78.15%
[34]	Chinese SL	Word	10	20,000	8	Armband placed on arm muscles: Same as the study published by the authors of [14]	200	10	98.66%
[35]	American SL	Sentence	250	10625	8	Armband placed on arm muscles: Same as the study published by the authors of [14]	200	15	WER 0.29%
[36]	American SL	Word	80	24,000	4	Extensor digitorum Flexor carpi radialis longus Extensor carpi radialis longus Extensor carpi ulnaris	1000	4	85.24–96.16%
[37]	American SL	Word	40	4000	4	Extensor digitorum Flexor carpi radialis longus Extensor carpi radialis longus Extensor carpi ulnaris	1000	4	95.94%
[38]	American SL	Word	27	2080	8	Armband placed on arm muscles: Same as the study published by the authors of [14]	200	1	From 60.85% to 80%
				10,400				10	From 34.00% to 51.54%
[39]	American SL	Word	70	ni	8	Armband placed on arm muscles: Same as the study published by the authors of [14]	200	15	93.7%
		Sentence	100						
[40]	American SL	Word	8	1300	8	Armband placed on arm muscles: Same as the study published by the authors of [14]	200	50	99.31%
		Alphabet	5						
[41]	American SL	Word	9	SCEPTRE database	8	Armband placed on arm muscles: Same as the study published by the authors of [14]	200	3	From 47.4% to 100%
[42]	American SL	Word	50	10,000	8	Armband placed on arm muscles: Same as the study published by the authors of [14]	200	10	33.66%
[43]	American SL	Word	20	4000	8	Armband placed on arm muscles: Same as the study published by the authors of [14]	200	20	97.9%
[44]	American SL	Digit	10	250	8	Armband placed on arm muscles: Same as the study published by the authors of [14]	1000	5	91.6 ± 3.5%
[45]	American SL	Alphabet	24	240	8	Armband placed on arm muscles: Same as the study published by the authors of [14]	200	1	80%
[46]	American SL	Word	13	390	8	Armband placed on arm muscles: Same as the study published by the authors of [14]	200	3	81.20%
[47]	American SL	Word	13	26,000	8	Armband placed on arm muscles: Same as the study published by the authors of [14]	200	3	93.79%
[48]	American SL	Hand shape	10	120	8 × 16	Intrinsic muscles Extrinsic muscles	400	4	78%
[49]	American SL	Alphabet	26	936	8	Armband placed on arm muscles: Same as the study published by the authors of [14]	200	8	95.36%
[50]	American SL	Alphabet	26	130	2	Flexor carpi radialis Extensor carpi radialis longus	-	1	95%
[51]	American SL	Word	10	3000	8	Armband placed on arm muscles: Same as the study published by the authors of [14]	200	-	95%
[52]	American SL	Alphabet	25	33,600	8	Armband placed on arm muscles: Same as the study published by the authors of [14]	200	7	100%
[53]	American SL	Alphabet	26	2080	8	-	960	1	92%
[54]	American SL	Word	20	-	8	Armband placed on arm muscles: Same as the study published by the authors of [14]	200	10	97.72%
[55]	American SL	Word	20	1800	6	-	2000	3	97%
[56]	Indian SL	Word	5	250	4	Flexor carpi radialis Extensor carpi radialis longus Reference electrode: Palm	1000	6	90%
[57]	Indian SL	Word	10	1200	3	Extensor carpi radialis longus Extensor digitorum Flexor carpi radialis	1111	6	87.5%
[58]	Indian SL	Digit/word	9	180	3	Where the maximum movement of the muscles of the forelimbs is observed	1000	-	91.1%
[59]	Indian SL	Word	100	16,000	3	-	1000	10	97%
[60]	Indian SL	Hand shape	15	1200	5	-	1111	-	100%
[61]	Indian SL	Hand shape + word	12	2400	2	Flexor digitorum Extensor carpi radialis	1111	10	88.25%
[62]	Indian SL	Digit	9	900	3	Flexor digitorum Extensor carpi radialis Brachioradialis	1111	5	90.10%
[63]	Indian SL	Hand shape	4	120	1	Flexor carpi radialis	1000	-	97.50%
[64]	Indian SL	Hand shape + word	10	800	3	Flexor capri ulnaris Extensor capri radialis Brachioradialis	1100	4	92.37%
[65]	Indian SL	Word	100	20,000	3	-	900	10	90.73%
[66]	Brazilian SL	Alphabet	20	2200	8	Armband placed on arm muscles: Same as the study published by the authors of [14]	200	1	From 4% to 95%
[67]	Brazilian SL	Alphabet	26	520	8	Armband placed on arm muscles: Same as the study published by the authors of [14]	200	10	89.11%
[68]	Brazilian SL	Alphabet	26	-	8	Armband placed on arm muscles: Same as the study published by the authors of [14]	200	15	99.06%
[69]	Brazilian SL	Alphabet	20	840	8	Extensor carpi ulnar Flexor carpi radial	200	1	81.60%
[70]	General hand shapes	Hand shapes	12	-	8	Brachial	200	-	-
[71]	General	Digit/alphabet	36	-	4	Lumbric muscles Hypothenar muscles Thenar muscles Flexor radials carpi	-	-	-
[72]	General	Hand shape	6	3600	8	Armband placed on arm muscles: Same as the study published by the authors of [14]	100	3	94%
[73]	General	Digit	10	500	3	-	-	-	86.80%
[74]	Indonesian SL	Word + alphabet	10	200	8	Armband placed on arm muscles: Same as [14]	200	1	98.63%
[75]	Indonesian SL	Alphabet	26	260	8	Armband placed on arm muscles: Same as the study published by the authors of [14]	200	1	93.08%
[76]	Indonesian SL	Word + alphabet	52	5200	8	Armband placed on arm muscles: Same as the study published by the authors of [14]	200	20	86.75%
[77]	Indonesian SL	Alphabet	26	260	8	Armband placed on arm muscles: Same as the study published by the authors of [14]	200	-	82.31%
[78]	Arabic SL	Alphabet	28	33,600	8	Armband placed on arm muscles: Same as the study published by the authors of [14]	200	3	98.49%
[79]	Arabic SL	Words	5	150	3	-	500	1	90.66%
[80]	Arabic SL	Alphabet	28	15,000	8	Armband placed on arm muscles: Same as the study published by the authors of [14]	200	8	97.4%
[81]	Italian SL	Alphabet	26	780	8	Armband placed on arm muscles: Same as the study published by the authors of [14]	200	30	97%
[82]	Italian SL	Alphabet		780	8	Armband placed on arm muscles: Same as the study published by the authors of [14]	200	1	93.5%
[83]	Italian SL	Alphabet	26	780	8	Armband placed on arm muscles: Same as the study published by the authors of [14]	200	1	-
[84]	Korean SL	Word	3	1200	8	Armband placed on arm muscles: Same as the study published by the authors of [14]	200	1	94%
[85]	Korean SL	Word	30	ni	8	Armband placed on arm muscles: Same as the study published by the authors of [14]	200	1	99.6%
[86]	Korean SL	Word	38	300	8	Armband placed on arm muscles: Same as the study published by the authors of [14], with the reference channel placed on the flexor carpi radialis	600	17	97.4%
[87]	Pakistani SL	Alphabet	26	780	3	Flexor carpi radialis Flexor digitorum superficialis		1	81%
[88]	Pakistani SL	Sentence	11	550	3	-	-	5	85.40%
[89]	Turkish SL	Number	11	1656	8	Armband placed on arm muscles: Same as the study published by the authors of [14]	200	9	86.61%
[90]	Turkish SL	Hand shape	36	-	8	-	200	10	78%
[91]	Malaysian SL	Word	5	150	1	Extensor carpi ulnaris	10	3	91%
[92]	Peru SL	Alphabet	27	135	4	-	100	1	93.9%
[93]	Polish SL	Word	18	21,420	8	Armband placed on arm muscles: Same as the study published by the authors of [14]	200	14	91%
[94]	German SL	Word	7	560	1	Flexor carpi radialis—nearby wrist	-	8	96.31%
[95]	French SL	Alphabet	7	2480	8	Armband placed on arm muscles: Same as the study published by the authors of [14]	200	4	90%
[96]	Persian SL	Word	20	2000	4	Extensor digitorum communis Flexor carpi radialis longus Extensor carpi radialis longus Extensor carpi ulnaris	-	10	96.13%
[97]	Colombian SL	Word	12	360	4	Extensor digitorum communis Extensor carpi ulnaris Flexor carpi ulnaris Flexor carpi radialis	1000	3	96.66%
[98]	Thai SL	Alphabet	10	2000	8	Armband placed on arm muscles: Same as the study published by the authors of [14]	1000	1	95%
[99]	Sinhala SL	Word	12	360	8	Armband placed on arm muscles: Same as the study published by the authors of [14]	200	6	94.4%
[100]	Irish SL	Alphabet	26	1560	8	Armband placed on arm muscles: Same as the study published by the authors of [14], with focus on the extensor digitorum from the posterior forearm, and the flexor carpi ulnaris from the anterior forearm	200	12	78%
[101]	Greek SL	Word	60	-	5	Flexor carpi ulnaris Flexor digitorum superficialis Flexor carpi radialis Extensor digitorum communis Extensor carpi ulnaris	1000	-	92%
